# Molecular pathways and emerging therapeutic targets in the pathogenesis of diabetic kidney disease

**DOI:** 10.3389/fphys.2026.1747053

**Published:** 2026-02-19

**Authors:** Sima Al-Masri, Jennifer N. Coelho, Linto Thomas

**Affiliations:** 1 Department of Molecular Pharmacology and Physiology, University of South Florida, Tampa, FL, United States; 2 Medical School, University of Sao Paulo, Sao Paulo, Brazil

**Keywords:** AGE–RAGE axis, AMP-activated protein kinase, diabetic kidney disease, DNA methylation, empagliflozin, epigenetics, fibrosis, finerenone

## Abstract

Diabetic kidney disease (DKD) arises from intersecting metabolic, hemodynamic, inflammatory, and epigenetic programs that progressively remodel the glomerulus and tubulointerstitium on a molecular level. Hyperglycemia-driven AGE-RAGE signaling, PKC activation, and RAAS dysregulation converge on oxidative stress, endothelial dysfunction, and profibrotic transcription (e.g., TGF-beta/Smad), while mitochondrial and endoplasmic-reticulum stress amplify lipotoxicity and cell death. Innate immune activation (macrophage recruitment and inflammasome signaling) and maladaptive repair promote extracellular-matrix accumulation and nephron loss. Multi-omics studies further implicate durable chromatin and non-coding RNA changes that sustain metabolic memory despite improved glycemia. In this review, we synthesize landmark and recent mechanistic data spanning glomerular filtration barrier injury, tubular stress pathways, and immune-metabolic crosstalk, and we highlight therapeutic strategies that move upstream of symptom control. We discuss established disease-modifying agents (RAAS blockade, SGLT2 inhibitors, and non-steroidal MR antagonists) alongside investigational approaches including epigenetic modulators, AMPK/NAD + axis targeting, and gene/RNA-based interventions. Together, these advances frame DKD as a disorder of rewired signaling and gene-regulatory circuitry, where convergent molecular nodes across podocytes, endothelium, and tubules offer the actionable considerations for durable renal protection.

## 1 Introduction

**FIGURE 1:**
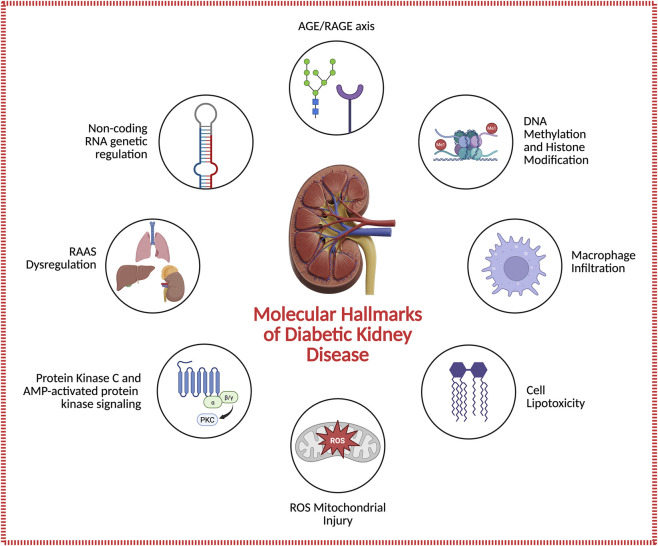
Molecular hallmarks of diabetic kidney disease (DKD). Chronic hyperglycemia triggers several interconnected pathways that drive renal injury and fibrosis. Shown are key mechanisms, including activation of the AGE/RAGE axis, dysregulation of the renin–angiotensin–aldosterone system (RAAS), abnormal protein kinase C (PKC) and AMP-activated protein kinase (AMPK) signaling, mitochondrial oxidative stress, lipid accumulation and lipotoxicity, macrophage infiltration, epigenetic alterations such as DNA methylation and histone modification, and non-coding RNA-mediated gene regulation. These processes collectively disrupt glomerular and tubular integrity, amplify inflammation, and promote fibrotic remodeling. The mechanisms illustrated represent major pathways but are not exhaustive of all molecular processes contributing to DKD pathogenesis.

Diabetic kidney disease (DKD) remains the leading cause of chronic kidney disease and end-stage renal failure worldwide, accounting for nearly half of new dialysis cases (Bakris et al., 2020; de Boer et al., 2022). Despite advances in glucose- and blood-pressure-lowering therapies, residual renal risk persists, underscoring the need for new mechanistic insight and targeted interventions. Chronic hyperglycemia triggers a network of maladaptive pathways, including the formation of advanced glycation end products (AGEs), activation of their receptor RAGE, and stimulation of protein kinase C (PKC) isoforms and the renin–angiotensin–aldosterone system (RAAS) that converge to promote oxidative stress, inflammation, and fibrosis (Neeper et al., 1992; Koya and King, 1998; Tanji et al., 2000; Wendt et al., 2003). Beyond metabolic and hemodynamic injury, organelle dysfunction, especially mitochondrial and endoplasmic-reticulum stress amplifies oxidative damage and apoptotic signaling (Victor et al., 2021; Wang and Zhang, 2024b). Multi-omics analyses have revealed epigenetic and transcriptional reprogramming that sustain these responses even after normoglycemia, producing a persistent “metabolic memory” (Smyth et al., 2022; Zheng et al., 2021). Clinically, RAAS blockade, sodium–glucose cotransporter 2 (SGLT2) inhibition, and non-steroidal mineralocorticoid receptor antagonism (ns-MRA) remain the therapeutic cornerstone yet only slow disease progression (Bakris et al., 2020; KDIGO, 2024). Preclinical and mechanistic studies support that SGLT2 inhibition can reduce diabetic glomerular hyperfiltration and may attenuate early albuminuria and renal hypertrophy (Dominguez Rieg et al., 2020). Emerging approaches, including epigenetic modulation, immunometabolic targeting, and gene- or RNA-based therapeutics, aim to interrupt upstream injury cascades and achieve durable renoprotection (Mimura et al., 2025; Li et al., 2025; Puri et al., 2025). This review synthesizes recent mechanistic and translational advances in DKD, highlighting the intersection of metabolic, hemodynamic, and epigenetic pathways and discussing how integrated molecular therapies may redefine kidney protection in diabetes. A schematic overview of these intersecting pathways is provided in Figure 1.

## 2 Core molecular mechanisms of diabetic kidney disease

### 2.1 Advanced glycation end products (AGEs) and RAGE activation

Advanced glycation end products (AGEs) are a heterogeneous group of molecules formed when reducing sugars, such as glucose, react non-enzymatically with proteins, lipids, or nucleic acids through the Maillard reaction. This glycation process alters the structure and function of biomolecules, making them stiffer, less soluble, and more prone to oxidative stress and inflammation.

The cloning and expression of the receptor for advanced glycation end products (RAGE) by Neeper and colleagues represented a critical advancement in the field of diabetic complications. Prior to this work, the molecular identity of the cell-surface receptor mediating the effects of AGEs was unknown. Neeper et al. (1992) isolated and sequenced RAGE, demonstrating it to be a member of the immunoglobulin superfamily with high affinity for AGEs and confirming its functional expression in mammalian cells. This discovery provided the essential molecular basis for subsequent research into the pathophysiological role of the AGE–RAGE axis in diabetes and its complications.

The identification of the receptor for advanced glycation end products (RAGE) enabled detailed studies of its expression in renal tissues, particularly in podocytes, and its upregulation in diabetic nephropathy. Tanji et al. (2000) demonstrated that RAGE is expressed on normal podocytes and is upregulated in diabetic nephropathy, with RAGE mRNA restricted to glomeruli and increased immunoreactivity correlating with the severity of glomerulosclerosis. This finding established the basis for subsequent mechanistic studies.

In animal models, accumulation of advanced glycation end products (AGEs) and upregulation of RAGE in renal tissues, especially in podocytes, lead to increased oxidative stress, inflammation, and activation of signaling pathways such as nuclear factor kappa B (NF-κB), mitogen-activated protein kinase (MAPK)/extracellular signal-regulated kinase (ERK), and phosphoinositide 3-kinase/protein kinase B (PI3K/Akt), resulting in glomerular injury, mesangial expansion, and albuminuria. Heidland et al. (2001), and Wendt et al. (2003) provided evidence that AGE-RAGE interaction in podocytes and mesangial cells enhances oxygen radical formation, activates NF-κB, and stimulates MAPK pathways, leading to pro-inflammatory cytokine release, growth factor induction, and glomerular structural changes. Chuang et al. (2007) further showed that AGE-RAGE signaling in podocytes activates p38MAPK and PI3K/Akt pathways, promoting apoptosis and glomerular injury.

Collectively, these studies support that RAGE identification enabled detailed investigation of its renal expression and pathogenic role in diabetic nephropathy, with upregulation in podocytes driving oxidative stress, inflammation, and signaling cascades that culminate in glomerular damage and albuminuria (Tanji et al., 2000; Heidland et al., 2001; Wendt et al., 2003; Chuang et al., 2007).

### 2.2 Protein kinase C (PKC) isoforms and downstream fibrotic signaling

Protein kinase C (PKC) isoforms, particularly PKC-β and PKC-α, are central mediators of fibrotic signaling in diabetic nephropathy and other microvascular complications. Hyperglycemia increases diacylglycerol (DAG) synthesis, which activates these PKC isoforms in renal tissues, leading to upregulation of profibrotic cytokines such as transforming growth factor-beta (TGF-β) and increased production of extracellular-matrix proteins including collagen and fibronectin. This cascade results in glomerular basement-membrane thickening and mesangial expansion, which are characteristic features of diabetic nephropathy (Koya et al., 1997; Koya and King, 1998; Whiteside and Dlugosz, 2002; Ohshiro et al., 2006; Wu et al., 2009).

PKC activation also enhances vascular permeability and inflammation by upregulating vascular endothelial growth factor (VEGF) and adhesion molecules, further contributing to tissue injury and fibrosis (Wu et al., 2009; Geraldes and King, 2010; Meier et al., 2009). Experimental models demonstrate that pharmacological inhibition of PKC-β (e.g., ruboxistaurin or LY333531) or dual inhibition of PKC-α and PKC-β can attenuate TGF-β expression, reduce extracellular-matrix accumulation, and decrease albuminuria, supporting the therapeutic potential of targeting PKC isoforms in diabetic kidney disease (Koya et al., 1997; Ohshiro et al., 2006; Meier et al., 2009; Menne et al., 2013).

In summary, PKC-β and PKC-α are the principal isoforms implicated in downstream fibrotic signaling in diabetic nephropathy, mediating TGF-β-driven extracellular-matrix production and vascular dysfunction. Inhibition of these isoforms has shown benefit in preclinical and early clinical studies, highlighting their role as therapeutic targets (Koya et al., 1997; Whiteside and Dlugosz, 2002; Ohshiro et al., 2006; Wu et al., 2009; Geraldes and King, 2010; Menne et al., 2013).

The latest clinical trial data indicate that PKC-β inhibitors, specifically ruboxistaurin, have demonstrated efficacy in reducing albuminuria, stabilizing estimated glomerular filtration rate (eGFR), and attenuating urinary TGF-β in patients with diabetic nephropathy already receiving optimal renin–angiotensin system blockade (Tuttle et al., 2005; Koya et al., 1997; Ohshiro et al., 2006). These effects have been consistently observed in phase 2 trials and secondary analyses of studies in diabetic retinopathy and neuropathy populations. Ruboxistaurin has shown a favorable safety profile, with no significant increase in adverse events compared with placebo at tested doses (Tuttle et al., 2005).

Dual inhibition of PKC-α and PKC-β isoforms has been shown in preclinical murine models to further reduce albuminuria, glomerular hypertrophy, and extracellular-matrix production, suggesting potential additive benefit, but clinical trial data in humans are currently lacking (Menne et al., 2013; Meier et al., 2009). In animal studies, dual inhibition was well tolerated, but human safety data are not yet available.

PKC-β inhibition remains investigational and is not yet standard of care; large-scale, prospective trials are needed to confirm long-term safety and efficacy in diabetic nephropathy (Koya et al., 1997; Ohshiro et al., 2006). No guideline from a major society currently recommends routine use of PKC-β or PKC-α inhibitors for diabetic nephropathy.

### 2.3 Renin–angiotensin–aldosterone system (RAAS) dysregulation

RAAS dysregulation is central to the pathogenesis and progression of diabetic kidney disease (DKD), driving glomerular hypertension, proteinuria, inflammation, and fibrosis. The classical RAAS axis, involving angiotensin II and aldosterone, promotes vasoconstriction, sodium retention, and fibrotic remodeling, while hyperglycemia-induced RAAS activation exacerbates oxidative stress and glomerular injury (Jia et al., 2024; Mende et al., 2023; Hughes et al., 2025). The RENAAL and IDNT landmark trials established angiotensin receptor blockers (losartan, irbesartan) as foundational therapies, demonstrating significant reductions in the risk of doubling serum creatinine, kidney failure, and death in patients with type 2 diabetes and albuminuric CKD (Brenner et al., 2001; Lewis et al., 2001).

Recent mechanistic studies highlight the role of mineralocorticoid receptor (MR) activation in mediating tubulointerstitial inflammation and fibrosis, with aldosterone acting *via* both MR-dependent and MR-independent pathways (Jia et al., 2024; Mende et al., 2023). The KDIGO (2024) guideline and the ADA–KDIGO consensus recommend ACE inhibitors or ARBs as first-line therapy for patients with diabetes, hypertension, and albuminuria, but caution against dual RAAS blockade due to increased risk of adverse events (de Boer et al., 2022; Michel et al., 2022). Two recent clinical studies have advanced the field: the FIDELIO-DKD trial demonstrated that the non-steroidal MR antagonist finerenone significantly reduced the risk of sustained eGFR decline, kidney failure, and cardiovascular events in DKD (Bakris et al., 2020; Sanglard et al., 2025; Chaudhuri et al., 2022; Tuttle et al., 2024). In mice, treatment with finerenone reversed kidney disease induced by a Western diet. Finerenone mitigated fibrosis (reducing fibronectin and collagen deposition) and inflammation (decreasing CD45^+^ leukocytes and CD68^+^ macrophages), while also preserving podocyte integrity and the glomerular glycocalyx layer. Its protective effects were linked to the regulation of lipid metabolism, the enhancement of mitochondrial oxidative phosphorylation (OXPHOS), and the increased expression of the nuclear receptor ERRγ (estrogen-related receptor gamma; Myakala et al., 2025). Additionally, a 2024 phase 2 trial of the aldosterone synthase inhibitor BI 690517 showed promising reductions in albuminuria and inflammation when added to standard RAAS inhibition, suggesting further therapeutic potential (Tuttle et al., 2024).

Emerging data also indicate that RAAS component levels vary with DKD complications and treatment, with aldosterone positively associated with insulin resistance and renal injury, underscoring the need for individualized RAAS-targeted therapy (Wang et al., 2025). Collectively, these findings support a paradigm in which RAAS modulation, primarily with ACE inhibitors, ARBs, and selective MR antagonists, remains the cornerstone of DKD management, with ongoing research into novel agents to address residual risk (de Boer et al., 2022; Chaudhuri et al., 2022; Michel et al., 2022).

### 2.4 Protein modifications and cellular stress responses

Diabetic kidney disease (DKD) is characterized by a convergence of metabolic, inflammatory, and hemodynamic insults that drive maladaptive protein modifications and cellular stress responses. Landmark mechanistic studies have established that chronic hyperglycemia induces non-enzymatic glycation of proteins, leading to the accumulation of advanced glycation end products (AGEs) and O-GlcNAcylation, which disrupt protein function and promote oxidative stress, inflammation, and fibrosis (Mohandes et al., 2023; Packer, 2023; Inagi, 2021). Multi-omics analyses have further revealed that aberrant protein phosphorylation and kinase signaling are critical in mediating glomerular and tubular injury, with dysregulated AMPK, PPAR, and HIF-1 pathways contributing to cellular dysfunction and fibrotic remodeling (Zhang et al., 2025a; Wei et al., 2024).

Recent studies have highlighted the interplay between endoplasmic reticulum (ER) stress and oxidative stress, showing that maladaptive unfolded protein response (UPR) activation in podocytes and tubular cells exacerbates apoptosis and inflammation, thereby accelerating DKD progression (Wang et al., 2022b; Victor et al., 2021; Wang et al., 2022a; Zhang et al., 2023). Notably, crosstalk between ER stress and mitochondrial dysfunction amplifies reactive oxygen species (ROS) production, further impairing cellular homeostasis (Wang and Zhang, 2024b). Clinical investigations have demonstrated that biomarkers of tubular injury and stress, such as kidney injury molecule-1 and cystatin C, rise early in DKD and correlate with disease severity (Lv et al., 2025).

Therapeutically, clinical trials targeting oxidative stress (e.g., bardoxolone methyl, an Nrf2 activator) and protein misfolding (e.g., chemical chaperones) have shown promise in slowing DKD progression, often in combination with renin–angiotensin–aldosterone system inhibitors and SGLT2 inhibitors (Zhang et al., 2025b; Wang and Zhang, 2024a). These findings underscore the importance of targeting protein modifications and cellular stress pathways for early diagnosis and intervention in DKD.

## 3 Epigenetic and transcriptional regulation in diabetic kidney disease

### 3.1 DNA methylation and histone remodeling

Epigenetic modifications, particularly DNA methylation and histone remodeling, are central to the pathogenesis and progression of diabetic kidney disease (DKD). Landmark integrative omics studies have demonstrated that persistent hyperglycemia induces stable changes in DNA methylation and histone marks, contributing to the “metabolic memory” phenomenon and sustained renal dysfunction even after glycemic normalization (Smyth et al., 2022; Li et al., 2023). Recent epigenome-wide meta-analyses have identified specific CpG methylation sites associated with DKD risk and progression, with several markers predicting kidney failure in both type 1 and type 2 diabetes cohorts (Smyth et al., 2022; Li K.Y. et al., 2023). Mechanistic studies have shown that aberrant DNA methyltransferase activity, such as DNMT3B, promotes renal fibrosis *via* Wnt/β-catenin pathway activation, and that histone modifications drive inflammation and extracellular matrix accumulation (Qu et al., 2025; Zheng et al., 2021).

### 3.2 Non-coding RNAs and regulatory networks

Non-coding RNAs, including microRNAs (miRNAs) and long non-coding RNAs (lncRNAs), orchestrate complex regulatory networks that modulate gene expression in DKD. Landmark studies have established that miRNAs such as miR-21, miR-192, and miR-155 exacerbate renal injury, while miR-126-3p and miR-29 confer protection (Rodzoń-Norwicz et al., 2025). Recent research has elucidated the crosstalk between lncRNAs and miRNAs, revealing bidirectional regulatory mechanisms that influence fibrosis, inflammation, and apoptosis in diabetic kidneys (Srivastava et al., 2021). Advances in RNA sequencing have identified stage-specific miRNA and lncRNA signatures, and clinical studies have demonstrated the utility of circulating and urinary miRNAs as non-invasive biomarkers for DKD diagnosis and prognosis (Rodzoń-Norwicz et al., 2025; Srivastava et al., 2021). Additionally, conventional therapies and dietary compounds may exert nephroprotective effects by modulating miRNA expression (Rodzoń-Norwicz et al., 2025).

### 3.3 Therapeutic targeting of epigenetic mechanisms

Therapeutic strategies targeting epigenetic mechanisms in DKD are rapidly evolving. Landmark preclinical studies have shown that pharmacological modulation of DNA methylation (e.g., 5-azacytidine, decitabine) and histone modifications (e.g., HDAC inhibitors, BET inhibitors) can attenuate renal injury and fibrosis (Martinez-Moreno et al., 2020; Fontecha-Barriuso et al., 2018). Recent clinical trials have evaluated the BD2-selective BET inhibitor apabetalone, which reached phase 3 for DKD with kidney function as a primary endpoint (Mimura et al., 2025; Akhouri et al., 2023). Additional studies have explored the nephroprotective effects of SGLT2 inhibitors, which may act *via* histone β-hydroxybutyrylation (Akhouri et al., 2023). While some epigenetic drugs are approved for malignancies, their application in DKD remains investigational, with ongoing research focused on safety, efficacy, and biomarker-driven patient selection (Akhouri et al., 2023). The reversibility of epigenetic memory offers a promising avenue for disease modification, but caution is warranted due to potential nephrotoxicity of some agents (Mimura et al., 2025; Akhouri et al., 2023).

## 4 Inflammation and fibrosis in DKD

### 4.1 Innate immunity and macrophage infiltration

Innate immune activation is a central driver of diabetic kidney disease (DKD), with macrophage infiltration and polarization playing pivotal roles in renal injury and disease progression (Ma et al., 2025; Fu et al., 2022; Li et al., 2022; Youssef et al., 2024; Zhang et al., 2025a; Zhao and Guo, 2025; Xu et al., 2023). Hyperglycemia and metabolic stress induce the release of damage-associated molecular patterns (DAMPs), which activate pattern recognition receptors such as Toll-like receptors and the NLRP3 inflammasome in resident renal cells, triggering a sterile inflammatory response (Tang and Yiu, 2020; Wan et al., 2021). This cascade leads to the recruitment of circulating monocytes, which differentiate into macrophages within the kidney. Single-cell transcriptomic analyses have revealed a dynamic shift in macrophage phenotypes, with early DKD characterized by increased M1-like proinflammatory macrophages that secrete cytokines such as TNF-α, IL-1β, and IL-6, amplifying local inflammation and tissue injury (Fu et al., 2022; Li et al., 2022; Youssef et al., 2024; Zhang et al., 2025a).

Over time, there is a transition toward M2-like anti-inflammatory and reparative macrophages, although this switch is often incomplete, contributing to persistent inflammation and fibrosis (Zhang et al., 2025a; Zhao and Guo, 2025; Xu et al., 2023). The renin–angiotensin system further modulates macrophage recruitment and polarization, and its pharmacologic blockade reduces macrophage infiltration and promotes M2 polarization, improving renal outcomes (Moratal et al., 2021). Key efferocytosis-related genes, including CD36, ITGAM, and CX3CR1, have been identified as critical modulators of macrophage function and are associated with proteinuria and renal dysfunction in DKD (Zhang et al., 2025b). Targeting macrophage activation, polarization, and cell–cell interactions represents a promising therapeutic strategy for attenuating DKD progression (Ma et al., 2025; Li et al., 2022; Youssef et al., 2024; Xu et al., 2023).

### 4.2 Crosstalk between inflammatory and fibrotic pathways

The progression of DKD is driven by intricate crosstalk between inflammatory and fibrotic signaling pathways, which together orchestrate glomerulosclerosis and tubulointerstitial fibrosis (Tang and Yiu, 2020; Wan et al., 2021; Ansari et al., 2025; Hou et al., 2025; Xu et al., 2023). Chronic hyperglycemia and oxidative stress activate proinflammatory cascades, including NF-κB, JAK–STAT, and the NLRP3 inflammasome, leading to sustained cytokine production and immune cell infiltration (Tang and Yiu, 2020; Wan et al., 2021). These inflammatory mediators stimulate renal cells (podocytes, mesangial cells, and tubular epithelial cells) to produce extracellular matrix (ECM) components and profibrotic factors such as TGF-β, driving fibroblast activation and epithelial–mesenchymal transition (EMT) (Tang and Yiu, 2020; Wan et al., 2021; Ansari et al., 2025; Xu et al., 2023).

The TGF-β/Smad, Wnt/β-catenin, PI3K/Akt, and MAPK pathways are central to the fibrotic response, promoting ECM deposition and irreversible tissue remodeling (Tang and Yiu, 2020; Wan et al., 2021). Macrophages serve as a nexus for this crosstalk, both amplifying inflammation and directly contributing to fibrosis through the secretion of profibrotic cytokines and by undergoing macrophage–myofibroblast transition (Li et al., 2022; Xu et al., 2023). The interplay between immune and metabolic signaling creates a feedback loop that accelerates kidney damage, with metabolic disturbances such as insulin resistance further exacerbating inflammatory and fibrotic responses (Hou et al., 2025). Emerging therapies, including SGLT2 inhibitors, GLP-1 receptor agonists, and novel immunomodulators, target these interconnected pathways, offering new avenues for disease modification (Hou et al., 2025; Ansari et al., 2025; Xu et al., 2023). Understanding the molecular mechanisms underlying this crosstalk is essential for developing targeted interventions to halt DKD progression.

## 5 Metabolic and oxidative stress pathways

### 5.1 Lipid accumulation and lipotoxicity in renal cells

Lipid accumulation and lipotoxicity in renal cells are central contributors to the pathogenesis and progression of diabetic kidney disease (DKD). Under diabetic conditions, renal cells, including podocytes, mesangial cells, and tubular epithelial cells, exhibit increased lipid uptake, impaired fatty acid oxidation, and disrupted cholesterol efflux, leading to intracellular accumulation of toxic lipid species such as free fatty acids, diacylglycerol, and ceramides (Opazo-Ríos et al., 2020; Thongnak et al., 2020; Gai et al., 2019; Ren et al., 2023; Yu et al., 2025; Chae et al., 2023). This ectopic lipid deposition triggers a cascade of cellular dysfunctions, including oxidative stress, mitochondrial dysfunction, endoplasmic reticulum stress, inflammation, and apoptosis, ultimately resulting in glomerular and tubular injury, fibrosis, and progressive loss of renal function (Opazo-Ríos et al., 2020; Ren et al., 2023; Yu et al., 2025; Hou et al., 2024; Han et al., 2021; Folestad et al., 2025).

Mechanistically, hyperglycemia-induced metabolic reprogramming and factors such as hypoxia, acting through hypoxia-inducible factor 1-alpha (HIF-1α), further suppress fatty acid oxidation and promote lipid synthesis, exacerbating lipid overload in renal cells (Yu et al., 2025; Chae et al., 2023; Hou et al., 2024). Lipotoxicity also impairs autophagic clearance of lipid droplets (lipophagy), amplifying lipid accumulation and tubular cell injury (Han et al., 2021). In addition, signaling pathways such as vascular endothelial growth factor B (VEGF-B)-mediated fatty acid transport from adipose tissue to the kidney have been implicated in glomerular lipid accumulation and correlate with renal dysfunction in both experimental models and human DKD (Folestad et al., 2025).

Collectively, these processes establish a feed-forward loop of metabolic, oxidative, and inflammatory injury that accelerates DKD progression. Targeting renal lipid metabolism and lipotoxicity is an emerging therapeutic strategy, with interventions aimed at restoring fatty acid oxidation, enhancing autophagy, and modulating lipid transport currently under active investigation (Yu et al., 2025; Chae et al., 2023; Ren et al., 2023; Hou et al., 2024; Han et al., 2021).

### 5.2 Mitochondrial dysfunction and bioenergetic failure

Mitochondrial dysfunction and bioenergetic failure are central to the pathogenesis and progression of diabetic kidney disease (DKD), particularly through exacerbating lipid accumulation and lipotoxicity in renal cells. In DKD, hyperglycemia and metabolic stress impair mitochondrial oxidative phosphorylation, leading to reduced ATP production and increased generation of mitochondrial reactive oxygen species (mtROS) (Hou et al., 2024; Ge et al., 2020; Ito et al., 2022; Ahmad et al., 2021; Ducasa et al., 2019; Yao et al., 2022; Hughes et al., 2025). This bioenergetic failure disrupts fatty acid oxidation, resulting in the accumulation of toxic lipid intermediates such as ceramides and diacylglycerol within podocytes, tubular epithelial cells, and mesangial cells (Ge et al., 2020; Hou et al., 2024; Yao et al., 2022).

The impaired mitochondrial function not only limits the capacity of renal cells to metabolize and clear excess lipids but also promotes lipid peroxidation and further mitochondrial damage, creating a vicious cycle of lipotoxicity and organelle dysfunction (Ge et al., 2020; Ducasa et al., 2019; Ren et al., 2023). Lipid overload triggers oxidative stress, endoplasmic reticulum stress, and inflammation, ultimately leading to apoptosis, tubular atrophy, and interstitial fibrosis (Opazo-Ríos et al., 2020; Chae et al., 2023).

Recent studies demonstrate that restoration of mitochondrial homeostasis, *via* agents such as the mitochondrial-targeted antioxidant SS-31 or recombinant Meteorin-like (Metrnl), can enhance fatty acid oxidation, reduce lipid accumulation, and attenuate renal injury in DKD models (Hou et al., 2024). In summary, mitochondrial dysfunction and bioenergetic failure drive lipid accumulation and lipotoxicity in renal cells by impairing fatty acid oxidation and promoting oxidative stress, thereby accelerating DKD progression (Hou et al., 2024; Ge et al., 2020; Ito et al., 2022; Ducasa et al., 2019; Yao et al., 2022; Hughes et al., 2025; Opazo-Ríos et al., 2020; Chae et al., 2023).

### 5.3 Reactive oxygen species (ROS) and oxidative injury

Oxidative stress, driven by excessive production of reactive oxygen species (ROS), is a central pathogenic mechanism in diabetic kidney disease (DKD). Landmark mechanistic studies have established that hyperglycemia activates multiple ROS-generating pathways, including mitochondrial dysfunction, NADPH oxidases, uncoupled endothelial nitric oxide synthase (eNOS), and xanthine oxidase, leading to cellular injury, inflammation, and fibrosis in renal tissues (Wang and Zhang, 2024b; Su et al., 2023; Darenskaya et al., 2023; Østergaard et al., 2020; Daehn et al., 2023; Jha et al., 2016; Chae et al., 2023; Sharma, 2016). Recent reviews highlight that ROS act as second messengers, modulating signaling cascades such as PI3K/Akt, TGF-β/p38-MAPK, and NF-κB, which perpetuate glomerular and tubular damage even after glycemic control is achieved (Jha et al., 2016).

Clinical and translational studies demonstrate that interventions targeting oxidative stress, such as the Nrf2 activator bardoxolone methyl, SGLT2 inhibitors, and GLP-1 receptor agonists, can slow DKD progression by reducing ROS burden and improving renal outcomes (Wang and Zhang, 2024a; Østergaard et al., 2020; Darenskaya et al., 2023). Additional antioxidant therapies, including lipoic acid and NADPH oxidase (Nox) inhibitors, are under investigation for their nephroprotective potential (Daehn et al., 2023; Chae et al., 2023). The persistence of oxidative injury despite optimal glucose control underscores the need for therapies that directly modulate ROS production and enhance endogenous antioxidant defenses (Su et al., 2023; Schiffer and Friederich-Persson, 2017).

### 5.4 AMP-activated protein kinase (AMPK) and redox balance

AMP-activated protein kinase (AMPK) is a master regulator of cellular energy and redox homeostasis, and its dysregulation is implicated in the pathogenesis of DKD. Landmark studies have demonstrated that AMPK activation mitigates oxidative stress, inflammation, and fibrosis by enhancing mitochondrial function, promoting autophagy, and suppressing pro-oxidant signaling (Hong et al., 2018; Li et al., 2021; Juszczak et al., 2020). Mechanistic evidence indicates that AMPK activation, *via* agents such as extracellular superoxide dismutase (EC-SOD), metformin, and small-molecule activators, improves renal function, reduces proteinuria, and attenuates fibrotic remodeling in experimental DKD models (Hong et al., 2018; Zhou et al., 2019; Salatto et al., 2017).

Clinical data suggest that selective activation of AMPK β1-containing isoforms leads to greater reductions in proteinuria and kidney injury markers than standard therapies, without significantly affecting glycemic control (Salatto et al., 2017). Furthermore, AMPK regulates the expression of antioxidant genes through the Nrf2 pathway and modulates lipid metabolism, thereby contributing to redox balance and renal protection (Juszczak et al., 2020; Daehn et al., 2023). The therapeutic potential of AMPK activation in DKD is supported by both preclinical and early clinical findings, with ongoing research focusing on improving pharmacologic specificity and delivery while minimizing off-target effects (Zhou et al., 2019; Li et al., 2021; Hong et al., 2018).

## 6 Therapeutic advances and future directions

### 6.1 SGLT2 inhibitors: benefits and limitations

Sodium-glucose cotransporter 2 (SGLT2) inhibitors provide substantial clinical benefits for patients with diabetic kidney disease (DKD), including marked reductions in the risk of kidney failure, end-stage renal disease, cardiovascular death, and hospitalization for heart failure, as demonstrated in large randomized controlled trials and meta-analyses (Natale et al., 2024; Bose et al., 2023; Mavrakanas et al., 2023; Brown et al., 2021; Kalantar-Zadeh et al., 2021). Current consensus reports from the American Diabetes Association (ADA) and Kidney Disease: Improving Global Outcomes (KDIGO) recommend SGLT2 inhibitors for most patients with type 2 diabetes and chronic kidney disease (CKD) with an estimated glomerular filtration rate (eGFR) ≥20 mL/min/1.73 m^2^, independent of glycemic control, due to their consistent cardiorenal protective effects (de Boer et al., 2022; KDIGO, 2024). These benefits are observed across a wide spectrum of baseline eGFR and albuminuria levels and extend to both diabetic and non-diabetic CKD populations (Brown et al., 2021; Kalantar-Zadeh et al., 2021; Mavrakanas et al., 2023). In addition, SGLT2 inhibitors carry a lower risk of hypoglycemia compared with other glucose-lowering agents and modestly reduce blood pressure and body weight (Natale et al., 2024; Bose et al., 2023). Mechanistically, SGLT2 inhibition decreases proximal tubular sodium–glucose reabsorption, increasing distal NaCl delivery and activating tubuloglomerular feedback to lower intraglomerular pressure and hyperfiltration (Dominguez Rieg et al., 2020). Compensatory SGLT1-mediated transport helps explain why SGLT2 blockade does not fully abolish fractional glucose reabsorption (Dominguez Rieg and Rieg, 2019).

Despite their favorable efficacy and safety profile, limitations exist. Glycemic efficacy diminishes at lower eGFR values, and an initial, reversible decline in eGFR may occur without requiring discontinuation (de Boer et al., 2022; KDIGO, 2024). Common adverse events include genital mycotic infections and volume depletion, with rare cases of diabetic ketoacidosis (Brown et al., 2021). Regular monitoring of renal function and hydration status is advised, especially in frail patients or those on diuretics. Because SGLT2 inhibitors alter proximal tubular transport, modest shifts in mineral handling (e.g., phosphate, urate, magnesium) and related hormones have been described and may warrant consideration in select patients (Dominguez Rieg et al., 2020; Thomas et al., 2022). SGLT2 inhibitors are not indicated in individuals receiving dialysis, and pediatric use remains limited to glycemic control (FDA, 2024; Burke et al., 2023).

Overall, SGLT2 inhibitors are now regarded as a cornerstone of DKD management. Their robust, evidence-based efficacy across diverse clinical settings demonstrates their role as key disease-modifying agents in the treatment of diabetic and non-diabetic CKD (Natale et al., 2024; Bose et al., 2023; Mavrakanas et al., 2023; KDIGO, 2024).

### 6.2 Emerging epigenetic and immunometabolic interventions

Recent advances in diabetic kidney disease (DKD) research highlight the therapeutic potential of targeting epigenetic and immunometabolic pathways that underlie persistent inflammation, oxidative stress, and fibrosis. These emerging strategies aim to reverse “metabolic memory” and immune dysregulation, which perpetuate renal injury even after glycemic normalization (Mimura et al., 2025; Bansal et al., 2020; Martinez-Moreno et al., 2020).

Epigenetic therapies focus on modulating DNA methylation, histone modifications, and non-coding RNAs to restore homeostatic gene expression. DNA methyltransferase (DNMT) inhibitors such as 5-azacytidine and decitabine have demonstrated renoprotective effects in preclinical DKD models by suppressing Wnt/β-catenin-driven fibrosis (Qu et al., 2025). Likewise, histone deacetylase (HDAC) inhibitors (e.g., valproate, trichostatin A) and BET inhibitors such as apabetalone reduce inflammation and extracellular matrix accumulation through inhibition of NF-κB and TGF-β pathways (Fontecha-Barriuso et al., 2018; Akhouri et al., 2023). Early-phase clinical trials of apabetalone have shown improved renal biomarkers and cardiovascular outcomes in diabetic populations, supporting the translational potential of epigenetic modulation in DKD (Martinez-Moreno et al., 2020).

Immunometabolic interventions target the metabolic–immune interface, where altered energy metabolism sustains chronic inflammation and macrophage polarization. Activating AMP-activated protein kinase (AMPK) or peroxisome proliferator-activated receptor-α (PPAR-α) enhances fatty-acid oxidation and suppresses pro-inflammatory cytokine release (Li et al., 2021; Juszczak et al., 2020). Similarly, SGLT2 inhibitors and GLP-1 receptor agonists exert epigenetic and immunometabolic benefits beyond glucose control, reducing oxidative stress, histone acetylation, and NLRP3 inflammasome activation in renal tissues (Brown et al., 2021; de Boer et al., 2022).

Emerging experimental approaches include NAD^+^ precursors (e.g., nicotinamide riboside) to enhance sirtuin activity, mitochondrial-targeted antioxidants to prevent epigenetic damage, and miRNA-based therapeutics to normalize aberrant gene regulation in DKD (Srivastava et al., 2021; Mimura et al., 2025). For example, treatment with nicotinamide riboside (NR) in diabetic db/db mice prevented manifestations of kidney dysfunction, such as albuminuria and mesangial expansion, effects associated with decreased inflammation. Mechanistically, NR increased the activity of the mitochondrial deacetylase SIRT3 and improved mitochondrial function, reducing mitochondrial DNA damage, which is a trigger for the activation of the cGAS-STING (cyclic GMP-AMP synthase-stimulator of interferon genes) pathway (Myakala et al., 2023). Collectively, these interventions reflect a paradigm shift toward disease-modifying therapies that integrate metabolic, immunologic, and epigenetic mechanisms to halt or reverse DKD progression.

### 6.3 Gene therapy and molecular editing tools

Gene therapy and molecular editing technologies represent a rapidly advancing frontier in the treatment of diabetic kidney disease (DKD), offering the possibility of precise and durable correction of pathogenic molecular pathways. Traditional pharmacologic interventions mitigate downstream injury, but gene-based strategies aim to directly modify or silence disease-driving genes, restore protective ones, and correct maladaptive signaling in renal cells (Liu et al., 2023; Sun et al., 2024).

RNA-based therapeutics, including small interfering RNA (siRNA), antisense oligonucleotides (ASOs), and microRNA (miRNA) mimics or inhibitors, have shown promise in modulating fibrosis, inflammation, and oxidative stress. For example, siRNA targeting transforming growth factor-β1 (TGF-β1) and connective tissue growth factor (CTGF) attenuates extracellular-matrix deposition and renal scarring in experimental DKD models (Wang et al., 2021). Inhibition of pro-fibrotic miR-21 or restoration of renoprotective miR-29 and miR-126-3p reduces mesangial expansion and improves glomerular function (Rodzoń-Norwicz et al., 2025; Srivastava et al., 2021).

CRISPR/Cas9 genome editing enables precise modification of key metabolic and inflammatory genes involved in DKD pathogenesis, including those encoding NADPH oxidase isoforms, SGLT2, and pro-fibrotic mediators such as SMAD3 and DNMT3B (Puri et al., 2025; Qu et al., 2025). Advances in CRISPR-based epigenome editing now allow targeted DNA methylation or histone acetylation changes without double-strand breaks, offering a safer route for reversible transcriptional reprogramming (Zheng et al., 2021). Similarly, base and prime editing systems have been used to correct single-nucleotide variants linked to diabetic susceptibility loci in podocytes and proximal tubular cells, highlighting their potential for personalized medicine (Liang et al., 2024).

To enhance renal specificity, recent studies employ kidney-targeted delivery systems such as lipid nanoparticles, viral vectors (AAV9, AAV-PHP.eB), and peptide-engineered exosomes that preferentially accumulate in glomerular and tubular compartments (Xie et al., 2023; Li et al., 2025). Combining these delivery platforms with inducible or cell-specific promoters may minimize off-target effects and immune responses. Preclinical data suggest that transient gene modulation, rather than permanent editing, may suffice to restore homeostasis and limit long-term risk.

Despite major advances, translation to clinical use remains limited by concerns about safety, Immunogenicity, scalability, and ethical regulation. Nevertheless, the convergence of high-fidelity CRISPR systems, RNA interference, and precision delivery technologies is expected to transform DKD therapy from symptomatic management to molecular correction. These tools provide an unprecedented opportunity to reprogram pathogenic renal phenotypes, opening a new era of regenerative nephrology (Sun et al., 2024; Liu et al., 2023).

### 6.4 Combination therapies and novel molecular targets

Combination therapies are increasingly recognized as the optimal approach for diabetic kidney disease (DKD), targeting multiple pathogenic pathways to achieve additive or synergistic cardiorenal protection. The Kidney Disease: Improving Global Outcomes (KDIGO) and American Diabetes Association (ADA) guidelines recommend a foundational regimen of renin-angiotensin system inhibitors (RASi), sodium–glucose cotransporter 2 inhibitors (SGLT2i), and non-steroidal mineralocorticoid receptor antagonists (ns-MRAs, e.g., finerenone) for most patients with DKD, with glucagon-like peptide-1 receptor agonists (GLP-1RA, e.g., semaglutide, dulaglutide) as adjuncts to improve glycemic and renal outcomes (Michel et al., 2022; Diabetes Care, 2025; Alicic et al., 2025; Mima, 2022; Georgianos et al., 2023; Khurana et al., 2022).

SGLT2 inhibitors (canagliflozin, dapagliflozin) and ns-MRAs (finerenone) exhibit complementary mechanisms, reducing glomerular hyperfiltration, inflammation, and fibrosis, while their combination significantly decreases albuminuria, slows eGFR decline, and reduces cardiovascular risk without excessive hyperkalemia (Alicic et al., 2025; Georgianos et al., 2023; Khurana et al., 2022; Martinez Leon et al., 2025). GLP-1RAs and dual GIP/GLP-1RAs (e.g., tirzepatide) further improve renal hemodynamics and mitigate oxidative and inflammatory stress, providing additional benefit beyond glycemic control (Wang and Zhang, 2024b; Li et al., 2021). Recent clinical trials and FDA approvals have broadened the therapeutic landscape to include endothelin receptor antagonists (e.g., atrasentan), aldosterone synthase inhibitors, and anti-inflammatory agents such as baricitinib and pentoxifylline, which target residual proteinuria, inflammation, and fibrosis (Nardone et al., 2025; Ghose et al., 2024; Mima, 2022). Also, studies comparing Sacubitril/Valsartan (Sac/Val) with Valsartan (Val) in type 2 diabetes models (db/db and KKAy mice) demonstrated that the combined therapy attenuated the progression of proteinuria, glomerulosclerosis, and podocyte loss. The self-DNA-activated cGAS-STING signaling pathway, identified as a new mechanism driving inflammation in diabetic kidneys, was activated in these models and prevented by both Sac/Val and Val treatment. Additionally, in db/db mice, Sac/Val improved mitochondrial DNA damage and Complex I activity and promoted fatty acid oxidation, suggesting that Sac/Val may enhance mitochondrial function compared to Val in this specific model (Myakala et al., 2021).

Novel molecular targets are also under investigation, including hypoxia-inducible factor (HIF) stabilizers, soluble guanylate cyclase (sGC) agonists, complement inhibitors, monoclonal antibodies against inflammatory mediators, and agents modulating oxidative stress and epigenetic pathways (Østergaard et al., 2020; Su et al., 2023; Huang et al., 2022).

By integrating hemodynamic, metabolic, inflammatory, and fibrotic mechanisms, multi-target therapy represents a paradigm shift in DKD management. Emerging clinical trials continue to optimize sequencing, safety, and efficacy of combination regimens to further reduce cardiorenal risk and improve long-term survival (Alicic et al., 2025; Nardone et al., 2025; Ghose et al., 2024). Key established and emerging therapies and their mechanistic targets are summarized in Table 1.

**TABLE 1:** Current and emerging DKD therapies and their mechanistic targets.

Pathway/Target	Representative agent(s)	Mechanistic action	Key evidence/Trials	Clinical status
Renin–angiotensin–aldosterone system (RAAS)	ACE inhibitors (enalapril), ARBs (losartan, irbesartan)	↓ Ang II–mediated vasoconstriction, fibrosis, and proteinuria	RENAAL (Brenner et al., 2001); IDNT (Lewis et al., 2001)	Standard of care
SGLT2 inhibition	Empagliflozin, Dapagliflozin, Canagliflozin	↓ Proximal tubular glucose/Na^+^ reabsorption → ↓ intraglomerular pressure; anti-inflammatory and antioxidant	DAPA-CKD (DAPA-CKD Trial Committees and Investigators, 2020); EMPA-KIDNEY (EMPA-KIDNEY Collaborative Group, 2022); CREDENCE (de Boer et al., 2022; Mavrakanas et al., 2023)	Approved cornerstone therapy
Non-steroidal mineralocorticoid receptor antagonists (ns-MRAs)	Finerenone	Anti-inflammatory and antifibrotic; ↓ TGF-β and collagen signaling	FIDELIO-DKD (Bakris et al., 2020); FIGARO-DKD (Pitt et al., 2021)	Approved for DKD
GLP-1 receptor agonists	Semaglutide, Dulaglutide, Tirzepatide	↓ Inflammation, oxidative stress, and weight; improves renal hemodynamics	REWIND (Li et al., 2021); SURPASS (Tanase et al., 2022)	Approved/expanding use
Combination multi-target therapy	RAAS + SGLT2i ± ns-MRA ± GLP-1RA	Synergistic reduction of albuminuria, fibrosis, and cardiovascular risk	Clinical data (Alicic et al., 2025; Georgianos et al., 2023; Michel et al., 2022)	Recommended standard strategy
AMPK activation/Immunometabolic regulation	Metformin, EC-SOD, small-molecule AMPK activators	Enhances mitochondrial function, autophagy, and redox balance	Preclinical and translational (Hong et al., 2018; Zhou et al., 2019; Li et al., 2021)	Clinical/preclinical
Oxidative-stress reduction (Nrf2, NOX)	Bardoxolone methyl, NOX inhibitors	Activates Nrf2 antioxidant response; ↓ ROS generation	BEACON/TSUBAKI trials (Østergaard et al., 2020; Su et al., 2023; Darenskaya et al., 2023)	Mixed results/ongoing
Aldosterone-synthase inhibition	BI 690517 (±empagliflozin)	↓ Aldosterone biosynthesis and MR activation	Phase 2 CKD trial (Tuttle et al., 2024)	Investigational
Protein kinase C (PKC-β) inhibition	Ruboxistaurin	Blocks DAG–PKC-β–TGF-β axis; ↓ ECM and albuminuria	Phase 2 trial (Tuttle et al., 2005); Mechanistic (Koya et al., 1997; Ohshiro et al., 2006)	Phase 2 complete/not marketed
Epigenetic modulation	5-Azacytidine, Decitabine, HDAC or BET inhibitors (Apabetalone)	Reverses metabolic memory *via* DNA/histone modification	Preclinical (Martinez-Moreno et al., 2020); Phase 3 (Mimura et al., 2025; Akhouri et al., 2023)	Investigational
Gene- and RNA-based therapies	siRNA (TGF-β1, CTGF), miRNA mimics/inhibitors, CRISPR-Cas9 edits	Direct modulation of fibrotic and inflammatory genes	Preclinical (Wang et al., 2021; Puri et al., 2025; Liang et al., 2024; Li et al., 2025)	Preclinical

## 7 Conclusion and outlook

Diabetic kidney disease (DKD) represents the cumulative effect of metabolic, hemodynamic, and epigenetic stressors that converge on shared inflammatory and fibrotic pathways. Decades of research have clarified the central roles of AGE–RAGE signaling, PKC activation, and RAAS dysregulation in driving oxidative injury and extracellular-matrix expansion (Tanji et al., 2000; Koya et al., 1997; Brenner et al., 2001). More recent work has expanded this view to include mitochondrial dysfunction, endoplasmic-reticulum stress, and maladaptive protein modification, which together perpetuate redox imbalance and cellular apoptosis (Victor et al., 2021; Wang and Zhang, 2024a).

Therapeutically, the integration of RAAS inhibitors, SGLT2 inhibitors, and non-steroidal mineralocorticoid receptor antagonists has transformed DKD management from glucose control to organ protection (Bakris et al., 2020; KDIGO, 2024). Yet substantial residual risk persists, emphasizing the need for combination and mechanism-targeted approaches that address metabolic memory and immunometabolic dysregulation. Emerging modalities, including epigenetic modifiers, AMPK activators, and RNA- or CRISPR-based interventions, offer opportunities to re-establish renal homeostasis rather than merely slow decline (Mimura et al., 2025; Puri et al., 2025).

Future work should prioritize biomarker-guided patient selection, synergistic multi-target regimens, and long-term safety evaluation across diverse populations. By coupling molecular precision with clinical integration, the next-generation of therapies may finally shift DKD treatment from supportive management to genuine disease modification.
